# (*E*)-1-(2-Hy­droxy-6-meth­oxy­phen­yl)-3-(2,4,6-tri­meth­oxy­phen­yl)prop-2-en-1-one

**DOI:** 10.1107/S1600536813031498

**Published:** 2013-11-23

**Authors:** Dongsoo Koh

**Affiliations:** a Department of Applied Chemistry, Dongduk Women’s University, Seoul 136-714, Republic of Korea

## Abstract

In the title mol­ecule, C_19_H_20_O_6_, the conformation about the C=C bond of the central enone group is *E*. The dihedral angle formed by the benzene rings is 11.6 (2)°. The hy­droxy group is involved in an intra­molecular O—H⋯O hydrogen bond. In the crystal, weak C—H⋯O hydrogen bonds link the mol­ecules into chains along [010].

## Related literature

For the synthesis and biological properties of chalcone deriv­atives, see: Shin *et al.* (2013 ▶); Yong *et al.* (2013 ▶); Hsieh *et al.* (2012 ▶); Sashidhara *et al.* (2011 ▶); Sharma *et al.* (2012 ▶). For related structures, see: Chantrapromma *et al.* (2013 ▶); Li *et al.* (2013 ▶). For standard bond lengths, see: Allen *et al.* (1987 ▶).
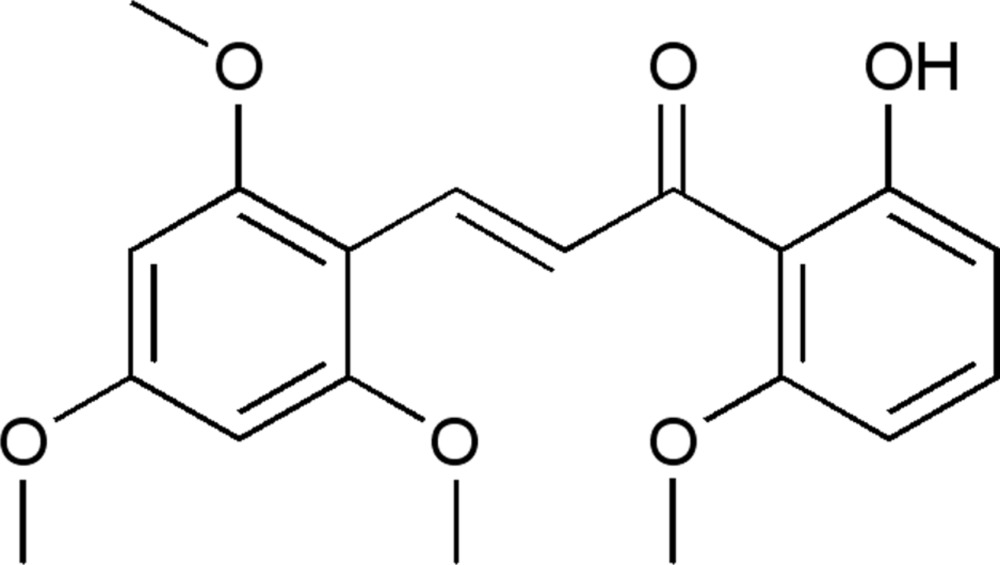


## Experimental

### 

#### Crystal data

C_19_H_20_O_6_
*M* *_r_* = 344.35
Monoclinic, 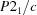

*a* = 7.2509 (11) Å
*b* = 15.670 (2) Å
*c* = 14.529 (2) Å
β = 99.579 (3)°
*V* = 1627.8 (4) Å^3^
*Z* = 4
Mo *K*α radiation
μ = 0.11 mm^−1^
*T* = 200 K
0.32 × 0.19 × 0.18 mm

#### Data collection

Bruker SMART CCD diffractometer
11993 measured reflections
4062 independent reflections
1641 reflections with *I* > 2σ(*I*)
*R*_int_ = 0.060

#### Refinement

*R*[*F* ^2^ > 2σ(*F* ^2^)] = 0.049
*wR*(*F* ^2^) = 0.146
*S* = 0.83
4062 reflections
231 parameters
H-atom parameters constrained
Δρ_max_ = 0.24 e Å^−3^
Δρ_min_ = −0.30 e Å^−3^

### 

Data collection: *SMART* (Bruker, 2000 ▶); cell refinement: *SAINT* (Bruker, 2000 ▶); data reduction: *SAINT*; program(s) used to solve structure: *SHELXS97* (Sheldrick, 2008 ▶); program(s) used to refine structure: *SHELXL97* (Sheldrick, 2008 ▶); molecular graphics: *SHELXTL* (Sheldrick, 2008 ▶); software used to prepare material for publication: *SHELXTL*.

## Supplementary Material

Crystal structure: contains datablock(s) I, New_Global_Publ_Block. DOI: 10.1107/S1600536813031498/lh5668sup1.cif

Structure factors: contains datablock(s) I. DOI: 10.1107/S1600536813031498/lh5668Isup2.hkl

Supplementary material file. DOI: 10.1107/S1600536813031498/lh5668Isup3.cml

Additional supplementary materials:  crystallographic information; 3D view; checkCIF report

## Figures and Tables

**Table 1:** Hydrogen-bond geometry (Å, °)

*D*—H⋯*A*	*D*—H	H⋯*A*	*D*⋯*A*	*D*—H⋯*A*
O5—H5⋯O1	0.84	1.73	2.475 (2)	147
C17—H17⋯O1^i^	0.95	2.42	3.265 (3)	148

Symmetry code: (i) 
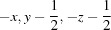
.

**Fig. 1.**
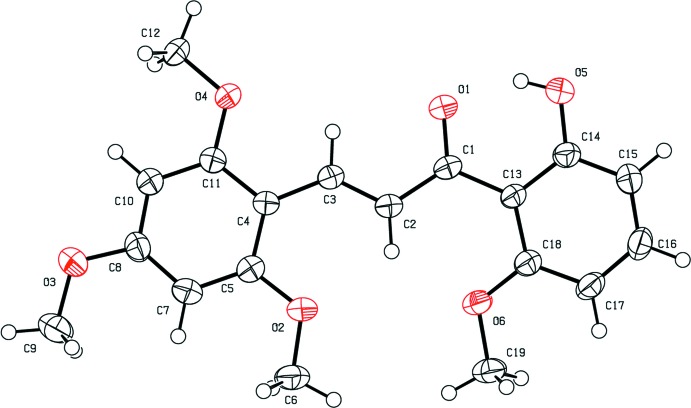
Molecular structure of the title compound, showing displacement ellipsoids drawn at the 50% probability level.

## supplementary crystallographic information

### 1. Comment

A variety of chlacones have been isolated from natural sources and synthesized, because they have shown wide spectrum of biological activities including anticancer (Shin *et al.*, 2013), anti-diabetic (Hsieh *et al.*, 2012), anti-inflammatory (Sashidhara *et al.* 2011), and antimicrobial (Sharma *et al.*, 2012). Chalcones are one of the secondary metabolites found in plants with a C6—C3—C6 skeleton and a C3 skeleton is an α,β-unsaturated carbonyl (enone). In continuation of our research interests to develop novel chalcones which show broad range of biological activities (Shin *et al.*, 2013, Yong *et al.*, 2013), the crystal structure of title compound has been determined.

The molecular structure of the title compound is shown in Fig. 1. The *trans* configuration of the C2═C3 double bond in the central enone group is confirmed by the dihedral angle of C1—C2═C3—C4 of 179.5 (2)°. The dihedral angle between the two benzene rings is 11.6 (2)°. The C1═O1 double bond [1.254 (3) Å] is slightly longer than the normal value (Allen *et al.* 1987) as this group is involved in an intramolecular hydrogen bond with the hydroxyl group. Of the three methoxy groups in the *B*-benzene ring, the two methoxy groups at the *ortho* positions are slightly more rotated from the ring plane (C6—O2—C5—C7 [9.7 (3)°], C12—O4—C11—C4 [172.8 (2)°]) than the group in the *para* position (C9—O3—C8—C10 [178.1 (2)°]). In the crystal, weak C—H···O hydrogen bonds link the molecules into one-dimensional chains along [010]. Examples of structures of substituted chalcone compounds have been published (Chantrapromma *et al.*, 2013; Li *et al.*, 2013).

### 2. Experimental

To a solution of 2-hydroxy-6-methoxyacetophenone (332 mg, 2 mmol) in 20 ml of anhydrous ethanol was added 2,4,6-trimethoxybenzaldehyde (392 mg, 2 mmol) and the temperature was adjusted to around 276–277 K in an ice-bath. To the cooled reaction mixture was added 2 ml of 50% aqueous KOH solution, and the reaction mixture was stirred at room temperature for 48 h. This mixture was poured into iced water (100 ml) and was acidified with 6 N HCl solution. The mixture was extracted with ethylacetate (3×20 ml) and the combined organic layers were dried under MgSO_4_. Filtration and evaporation of the filtrate gave a residue which was purified by flash chromatography to afford the title compound (255 mg, 34%). Recrystallization of the title compound in ethanol gave single crystals (mp: 401–402 K).

### 3. Refinement

H atoms were placed in calculated positions and refined as riding with C—H = 0.95–0.98 Å, O—H = 0.84 Å and *U*_iso_(H) = 1.2 *U*_eq_(C) or *U*_iso_(H) = 1.5*U*_eq_(C_methyl_, O).

### Crystal data

**Table:**

C_19_H_20_O_6_	*F*(000) = 728
*M**_r_* = 344.35	*D*_x_ = 1.405 Mg m^−^^3^
Monoclinic, *P*2_1_/*c*	Mo *K*α radiation, λ = 0.71073 Å
Hall symbol: -P 2ybc	Cell parameters from 2416 reflections
*a* = 7.2509 (11) Å	θ = 2.6–26.0°
*b* = 15.670 (2) Å	µ = 0.11 mm^−^^1^
*c* = 14.529 (2) Å	*T* = 200 K
β = 99.579 (3)°	Block, yellow
*V* = 1627.8 (4) Å^3^	0.32 × 0.19 × 0.18 mm
*Z* = 4	

### Data collection

**Table:**

Bruker SMART CCD diffractometer	1641 reflections with *I* > 2σ(*I*)
Radiation source: fine-focus sealed tube	*R*_int_ = 0.060
Graphite monochromator	θ_max_ = 28.4°, θ_min_ = 1.9°
φ and ω scans	*h* = −9→9
11993 measured reflections	*k* = −20→17
4062 independent reflections	*l* = −12→19

### Refinement

**Table:**

Refinement on *F*^2^	Primary atom site location: structure-invariant direct methods
Least-squares matrix: full	Secondary atom site location: difference Fourier map
*R*[*F*^2^ > 2σ(*F*^2^)] = 0.049	Hydrogen site location: inferred from neighbouring sites
*wR*(*F*^2^) = 0.146	H-atom parameters constrained
*S* = 0.83	*w* = 1/[σ^2^(*F*_o_^2^) + (0.0623*P*)^2^] where *P* = (*F*_o_^2^ + 2*F*_c_^2^)/3
4062 reflections	(Δ/σ)_max_ < 0.001
231 parameters	Δρ_max_ = 0.24 e Å^−^^3^
0 restraints	Δρ_min_ = −0.30 e Å^−^^3^

### Special details

**Table:**

Geometry. All e.s.d.'s (except the e.s.d. in the dihedral angle between two l.s. planes) are estimated using the full covariance matrix. The cell e.s.d.'s are taken into account individually in the estimation of e.s.d.'s in distances, angles and torsion angles; correlations between e.s.d.'s in cell parameters are only used when they are defined by crystal symmetry. An approximate (isotropic) treatment of cell e.s.d.'s is used for estimating e.s.d.'s involving l.s. planes.
Refinement. Refinement of *F*^2^ against ALL reflections. The weighted *R*-factor *wR* and goodness of fit *S* are based on *F*^2^, conventional *R*-factors *R* are based on *F*, with *F* set to zero for negative *F*^2^. The threshold expression of *F*^2^ > σ(*F*^2^) is used only for calculating *R*-factors(gt) *etc*. and is not relevant to the choice of reflections for refinement. *R*-factors based on *F*^2^ are statistically about twice as large as those based on *F*, and *R*- factors based on ALL data will be even larger.

### Fractional atomic coordinates and isotropic or equivalent isotropic displacement parameters (Å^2^)

**Table:**

	*x*	*y*	*z*	*U*_iso_*/*U*_eq_	
C1	0.1235 (3)	0.18277 (14)	−0.17106 (17)	0.0341 (6)	
O1	0.0997 (2)	0.25771 (9)	−0.20093 (12)	0.0470 (5)	
C2	0.2322 (3)	0.16860 (14)	−0.07822 (16)	0.0348 (6)	
H2	0.2615	0.1122	−0.0568	0.042*	
C3	0.2905 (3)	0.23561 (14)	−0.02317 (17)	0.0342 (6)	
H3	0.2559	0.2896	−0.0504	0.041*	
C4	0.3967 (3)	0.23973 (13)	0.07012 (16)	0.0310 (6)	
C5	0.4563 (3)	0.16888 (14)	0.12690 (17)	0.0349 (6)	
O2	0.4201 (2)	0.09143 (9)	0.08508 (11)	0.0442 (5)	
C6	0.4501 (4)	0.01601 (14)	0.14069 (19)	0.0481 (7)	
H6A	0.5830	0.0115	0.1676	0.072*	
H6B	0.4125	−0.0340	0.1016	0.072*	
H6C	0.3755	0.0188	0.1910	0.072*	
C7	0.5470 (3)	0.17725 (15)	0.21872 (17)	0.0386 (6)	
H7	0.5846	0.1282	0.2556	0.046*	
C8	0.5812 (3)	0.25842 (15)	0.25526 (17)	0.0377 (6)	
O3	0.6695 (2)	0.27612 (10)	0.34409 (12)	0.0483 (5)	
C9	0.7234 (4)	0.20637 (16)	0.40476 (18)	0.0547 (8)	
H9A	0.6125	0.1731	0.4123	0.082*	
H9B	0.7838	0.2278	0.4657	0.082*	
H9C	0.8112	0.1700	0.3782	0.082*	
C10	0.5288 (3)	0.33007 (14)	0.20224 (17)	0.0380 (6)	
H10	0.5550	0.3853	0.2280	0.046*	
C11	0.4386 (3)	0.32097 (14)	0.11176 (16)	0.0339 (6)	
O4	0.3845 (2)	0.38954 (9)	0.05574 (11)	0.0415 (5)	
C12	0.4042 (4)	0.47228 (13)	0.09820 (18)	0.0433 (7)	
H12A	0.3354	0.4740	0.1508	0.065*	
H12B	0.3538	0.5156	0.0521	0.065*	
H12C	0.5369	0.4839	0.1207	0.065*	
C13	0.0480 (3)	0.11226 (13)	−0.23419 (16)	0.0327 (6)	
C14	−0.0110 (3)	0.13084 (14)	−0.32983 (17)	0.0354 (6)	
O5	−0.0123 (3)	0.21060 (10)	−0.36318 (11)	0.0456 (5)	
H5	0.0250	0.2445	−0.3192	0.068*	
C15	−0.0686 (3)	0.06696 (15)	−0.39475 (17)	0.0401 (6)	
H15	−0.1026	0.0807	−0.4590	0.048*	
C16	−0.0759 (3)	−0.01563 (15)	−0.36553 (18)	0.0416 (7)	
H16	−0.1145	−0.0591	−0.4102	0.050*	
C17	−0.0285 (3)	−0.03734 (14)	−0.27249 (18)	0.0394 (6)	
H17	−0.0370	−0.0950	−0.2534	0.047*	
C18	0.0315 (3)	0.02531 (13)	−0.20719 (17)	0.0337 (6)	
O6	0.0748 (2)	0.00753 (9)	−0.11397 (11)	0.0433 (5)	
C19	0.0704 (4)	−0.07976 (14)	−0.08470 (18)	0.0453 (7)	
H19A	−0.0563	−0.1025	−0.1028	0.068*	
H19B	0.1065	−0.0830	−0.0167	0.068*	
H19C	0.1580	−0.1134	−0.1145	0.068*	

### Atomic displacement parameters (Å^2^)

**Table:**

	*U*^11^	*U*^22^	*U*^33^	*U*^12^	*U*^13^	*U*^23^
C1	0.0405 (15)	0.0230 (12)	0.0382 (15)	0.0022 (10)	0.0044 (12)	−0.0002 (11)
O1	0.0698 (13)	0.0252 (9)	0.0408 (11)	−0.0004 (8)	−0.0057 (9)	0.0021 (8)
C2	0.0411 (15)	0.0252 (12)	0.0363 (15)	0.0009 (11)	0.0010 (12)	0.0031 (11)
C3	0.0393 (15)	0.0252 (12)	0.0358 (15)	−0.0015 (10)	−0.0005 (12)	0.0015 (11)
C4	0.0351 (14)	0.0264 (12)	0.0305 (14)	0.0018 (10)	0.0027 (11)	0.0023 (10)
C5	0.0375 (15)	0.0302 (13)	0.0370 (15)	−0.0002 (11)	0.0059 (12)	−0.0011 (11)
O2	0.0609 (12)	0.0265 (9)	0.0422 (11)	0.0026 (8)	−0.0008 (9)	0.0057 (8)
C6	0.0554 (18)	0.0306 (14)	0.0558 (18)	0.0047 (12)	0.0019 (15)	0.0102 (13)
C7	0.0412 (16)	0.0349 (13)	0.0384 (16)	0.0049 (11)	0.0027 (12)	0.0068 (12)
C8	0.0378 (15)	0.0411 (15)	0.0315 (15)	0.0011 (11)	−0.0020 (12)	−0.0014 (12)
O3	0.0615 (13)	0.0421 (10)	0.0366 (11)	0.0061 (9)	−0.0054 (10)	0.0035 (9)
C9	0.071 (2)	0.0501 (17)	0.0406 (17)	0.0101 (15)	0.0033 (15)	0.0119 (14)
C10	0.0449 (16)	0.0313 (13)	0.0350 (15)	0.0016 (11)	−0.0013 (12)	−0.0009 (11)
C11	0.0375 (15)	0.0302 (13)	0.0324 (14)	0.0023 (11)	0.0011 (12)	0.0008 (11)
O4	0.0581 (12)	0.0245 (9)	0.0371 (10)	0.0012 (8)	−0.0060 (9)	−0.0023 (7)
C12	0.0537 (17)	0.0259 (13)	0.0454 (16)	−0.0018 (11)	−0.0060 (13)	−0.0065 (11)
C13	0.0372 (14)	0.0229 (12)	0.0354 (14)	0.0013 (10)	−0.0011 (11)	−0.0018 (10)
C14	0.0349 (15)	0.0276 (13)	0.0408 (16)	0.0012 (10)	−0.0018 (12)	0.0014 (12)
O5	0.0611 (13)	0.0325 (9)	0.0384 (11)	−0.0022 (8)	−0.0055 (9)	0.0050 (8)
C15	0.0433 (16)	0.0399 (15)	0.0340 (15)	0.0004 (12)	−0.0026 (12)	−0.0026 (12)
C16	0.0434 (16)	0.0380 (15)	0.0407 (16)	−0.0013 (12)	−0.0009 (13)	−0.0131 (13)
C17	0.0442 (16)	0.0250 (12)	0.0478 (17)	−0.0009 (11)	0.0042 (13)	−0.0060 (12)
C18	0.0363 (14)	0.0278 (13)	0.0365 (15)	0.0006 (10)	0.0045 (12)	−0.0013 (11)
O6	0.0681 (13)	0.0248 (9)	0.0366 (11)	−0.0036 (8)	0.0079 (9)	0.0018 (8)
C19	0.0591 (18)	0.0279 (13)	0.0495 (17)	−0.0022 (12)	0.0105 (14)	0.0075 (12)

### Geometric parameters (Å, º)

**Table:**

C1—O1	1.254 (2)	C10—C11	1.375 (3)
C1—C2	1.461 (3)	C10—H10	0.9500
C1—C13	1.481 (3)	C11—O4	1.365 (2)
C2—C3	1.345 (3)	O4—C12	1.433 (2)
C2—H2	0.9500	C12—H12A	0.9800
C3—C4	1.445 (3)	C12—H12B	0.9800
C3—H3	0.9500	C12—H12C	0.9800
C4—C5	1.408 (3)	C13—C14	1.414 (3)
C4—C11	1.420 (3)	C13—C18	1.428 (3)
C5—O2	1.363 (2)	C14—O5	1.340 (2)
C5—C7	1.392 (3)	C14—C15	1.391 (3)
O2—C6	1.428 (3)	O5—H5	0.8400
C6—H6A	0.9800	C15—C16	1.366 (3)
C6—H6B	0.9800	C15—H15	0.9500
C6—H6C	0.9800	C16—C17	1.381 (3)
C7—C8	1.385 (3)	C16—H16	0.9500
C7—H7	0.9500	C17—C18	1.384 (3)
C8—O3	1.370 (3)	C17—H17	0.9500
C8—C10	1.379 (3)	C18—O6	1.367 (3)
O3—C9	1.418 (3)	O6—C19	1.434 (2)
C9—H9A	0.9800	C19—H19A	0.9800
C9—H9B	0.9800	C19—H19B	0.9800
C9—H9C	0.9800	C19—H19C	0.9800
			
O1—C1—C2	118.9 (2)	C8—C10—H10	120.2
O1—C1—C13	118.0 (2)	O4—C11—C10	122.1 (2)
C2—C1—C13	123.03 (19)	O4—C11—C4	115.58 (19)
C3—C2—C1	119.9 (2)	C10—C11—C4	122.3 (2)
C3—C2—H2	120.1	C11—O4—C12	117.22 (18)
C1—C2—H2	120.1	O4—C12—H12A	109.5
C2—C3—C4	131.2 (2)	O4—C12—H12B	109.5
C2—C3—H3	114.4	H12A—C12—H12B	109.5
C4—C3—H3	114.4	O4—C12—H12C	109.5
C5—C4—C11	115.7 (2)	H12A—C12—H12C	109.5
C5—C4—C3	125.3 (2)	H12B—C12—H12C	109.5
C11—C4—C3	118.84 (19)	C14—C13—C18	116.1 (2)
O2—C5—C7	122.4 (2)	C14—C13—C1	118.37 (19)
O2—C5—C4	115.1 (2)	C18—C13—C1	125.5 (2)
C7—C5—C4	122.5 (2)	O5—C14—C15	116.3 (2)
C5—O2—C6	119.01 (19)	O5—C14—C13	122.0 (2)
O2—C6—H6A	109.5	C15—C14—C13	121.7 (2)
O2—C6—H6B	109.5	C14—O5—H5	109.5
H6A—C6—H6B	109.5	C16—C15—C14	119.6 (2)
O2—C6—H6C	109.5	C16—C15—H15	120.2
H6A—C6—H6C	109.5	C14—C15—H15	120.2
H6B—C6—H6C	109.5	C15—C16—C17	121.4 (2)
C8—C7—C5	118.7 (2)	C15—C16—H16	119.3
C8—C7—H7	120.7	C17—C16—H16	119.3
C5—C7—H7	120.7	C16—C17—C18	119.7 (2)
O3—C8—C10	113.8 (2)	C16—C17—H17	120.2
O3—C8—C7	125.0 (2)	C18—C17—H17	120.2
C10—C8—C7	121.2 (2)	O6—C18—C17	121.9 (2)
C8—O3—C9	117.82 (19)	O6—C18—C13	116.8 (2)
O3—C9—H9A	109.5	C17—C18—C13	121.3 (2)
O3—C9—H9B	109.5	C18—O6—C19	118.38 (17)
H9A—C9—H9B	109.5	O6—C19—H19A	109.5
O3—C9—H9C	109.5	O6—C19—H19B	109.5
H9A—C9—H9C	109.5	H19A—C19—H19B	109.5
H9B—C9—H9C	109.5	O6—C19—H19C	109.5
C11—C10—C8	119.5 (2)	H19A—C19—H19C	109.5
C11—C10—H10	120.2	H19B—C19—H19C	109.5
			
O1—C1—C2—C3	5.9 (4)	C3—C4—C11—C10	175.9 (2)
C13—C1—C2—C3	−177.5 (2)	C10—C11—O4—C12	−7.6 (3)
C1—C2—C3—C4	179.5 (2)	C4—C11—O4—C12	172.9 (2)
C2—C3—C4—C5	−3.3 (4)	O1—C1—C13—C14	13.0 (3)
C2—C3—C4—C11	−179.9 (3)	C2—C1—C13—C14	−163.5 (2)
C11—C4—C5—O2	−178.1 (2)	O1—C1—C13—C18	−167.9 (2)
C3—C4—C5—O2	5.2 (3)	C2—C1—C13—C18	15.5 (4)
C11—C4—C5—C7	1.3 (3)	C18—C13—C14—O5	176.3 (2)
C3—C4—C5—C7	−175.3 (2)	C1—C13—C14—O5	−4.6 (4)
C7—C5—O2—C6	9.7 (3)	C18—C13—C14—C15	−4.6 (4)
C4—C5—O2—C6	−170.9 (2)	C1—C13—C14—C15	174.5 (2)
O2—C5—C7—C8	178.8 (2)	O5—C14—C15—C16	−178.2 (2)
C4—C5—C7—C8	−0.6 (4)	C13—C14—C15—C16	2.7 (4)
C5—C7—C8—O3	−179.7 (2)	C14—C15—C16—C17	0.4 (4)
C5—C7—C8—C10	−0.6 (4)	C15—C16—C17—C18	−1.3 (4)
C10—C8—O3—C9	178.1 (2)	C16—C17—C18—O6	178.0 (2)
C7—C8—O3—C9	−2.7 (4)	C16—C17—C18—C13	−0.9 (4)
O3—C8—C10—C11	−179.9 (2)	C14—C13—C18—O6	−175.3 (2)
C7—C8—C10—C11	0.9 (4)	C1—C13—C18—O6	5.7 (4)
C8—C10—C11—O4	−179.6 (2)	C14—C13—C18—C17	3.7 (4)
C8—C10—C11—C4	−0.1 (4)	C1—C13—C18—C17	−175.3 (2)
C5—C4—C11—O4	178.5 (2)	C17—C18—O6—C19	4.6 (3)
C3—C4—C11—O4	−4.6 (3)	C13—C18—O6—C19	−176.4 (2)
C5—C4—C11—C10	−1.0 (3)		

### Hydrogen-bond geometry (Å, º)

**Table:**

*D*—H···*A*	*D*—H	H···*A*	*D*···*A*	*D*—H···*A*
O5—H5···O1	0.84	1.73	2.475 (2)	147
C17—H17···O1^i^	0.95	2.42	3.265 (3)	148

Symmetry code: (i) −*x*, *y*−1/2, −*z*−1/2.

## References

[bb1] Allen, F. H., Kennard, O., Watson, D. G., Brammer, L., Orpen, A. G. & Taylor, R. (1987). *J. Chem. Soc. Perkin Trans. 2*, pp. S1–19.

[bb2] Bruker (2000). *SMART* and *SAINT* Bruker AXS Inc., Madison, Wisconsin, USA.

[bb3] Chantrapromma, S., Ruanwas, P., Boonnak, N. & Fun, H.-K. (2013). *Acta Cryst.* E**69**, o1004–o1005. 10.1107/S1600536813014189 PMC3772449 24046592

[bb4] Hsieh, C.-T., Hsieh, T.-J., El-Shazly, M., Chuang, D.-W., Tsai, Y.-H., Yen, C.-T., Wu, S.-F., Wu, Y.-C. & Chang, F.-R. (2012). *Bioorg. Med. Chem. Lett.* **22**, 3912–3915. 10.1016/j.bmcl.2012.04.108 22608392

[bb5] Li, R., Li, D.-D. & Wu, J.-Y. (2013). *Acta Cryst.* E**69**, o1405. 10.1107/S1600536813021946 PMC3884491 24427043

[bb6] Sashidhara, K. V., Kumar, M., Modukuri, R. M., Sonkar, R., Bhatia, G., Khanna, A. K., Rai, S. V. & Shukla, R. (2011). *Bioorg. Med. Chem. Lett.* **21**, 4480–4484. 10.1016/j.bmcl.2011.06.002 21723119

[bb7] Sharma, V., Singh, G., Kaur, H., Saxena, A. K. & Ishar, M. P. S. (2012). *Bioorg. Med. Chem. Lett.* **22**, 6343–6346. 10.1016/j.bmcl.2012.08.084 22999415

[bb8] Sheldrick, G. M. (2008). *Acta Cryst.* A**64**, 112–122. 10.1107/S0108767307043930 18156677

[bb9] Shin, S. Y., Yoon, H., Hwang, D., Ahn, S., Kim, D.-W., Koh, D., Lee, Y. H. & Lim, Y. (2013). *Bioorg. Med. Chem.* **21**, 7018–7024. 10.1016/j.bmc.2013.09.014 24095020

[bb10] Yong, Y., Ahn, S., Hwang, D., Yoon, H., Jo, G., Kim, Y. H., Kim, S. H., Koh, D. & Lim, Y. (2013). *Magn. Reson. Chem.* **51**, 364–370. 10.1002/mrc.3949 23592179

